# Contagion Management at the Méditerranée Infection University Hospital Institute

**DOI:** 10.3390/jcm10122627

**Published:** 2021-06-15

**Authors:** Pierre-Edouard Fournier, Sophie Edouard, Nathalie Wurtz, Justine Raclot, Marion Bechet, Christine Zandotti, Véronique Filosa, Didier Raoult, Florence Fenollar

**Affiliations:** 1 IHU-Méditerranée Infection, 19–21 Boulevard Jean Moulin, 13005 Marseille, France; Pierre-edouard.fournier@univ-amu.fr (P.-E.F.); Sophie.edouard@ap-hm.fr (S.E.); nathalie.wurtz@univ-amu.fr (N.W.); justine.raclot@ap-hm.fr (J.R.); marion.bechet@ap-hm.fr (M.B.); christine.zandotti@ap-hm.fr (C.Z.); veronique.filosa@ap-hm.fr (V.F.); didier.raoult@gmail.com (D.R.); 2 VITROME Unit, IRD, AP-HM, SSA, IHU-Méditerranée Infection, Aix Marseille University, 13005 Marseille, France; 3 MEPHI Unit, IRD, AP-HM, IHU Méditerranée Infection, Aix Marseille University, 13005 Marseille, France

**Keywords:** contagion, institut hospitalo-universitaire méditerranée infection, SARS-CoV-2, COVID-19, diagnosis, rapid diagnostic test, point-of-care

## Abstract

The Méditerranée Infection University Hospital Institute (IHU) is located in a recent building, which includes experts on a wide range of infectious disease. The IHU strategy is to develop innovative tools, including epidemiological monitoring, point-of-care laboratories, and the ability to mass screen the population. In this study, we review the strategy and guidelines proposed by the IHU and its application to the COVID-19 pandemic and summarise the various challenges it raises. Early diagnosis enables contagious patients to be isolated and treatment to be initiated at an early stage to reduce the microbial load and contagiousness. In the context of the COVID-19 pandemic, we had to deal with a shortage of personal protective equipment and reagents and a massive influx of patients. Between 27 January 2020 and 5 January 2021, 434,925 nasopharyngeal samples were tested for the presence of SARS-CoV-2. Of them, 12,055 patients with COVID-19 were followed up in our out-patient clinic, and 1888 patients were hospitalised in the Institute. By constantly adapting our strategy to the ongoing situation, the IHU has succeeded in expanding and upgrading its equipment and improving circuits and flows to better manage infected patients.

## 1. Introduction

**Figure 1:**
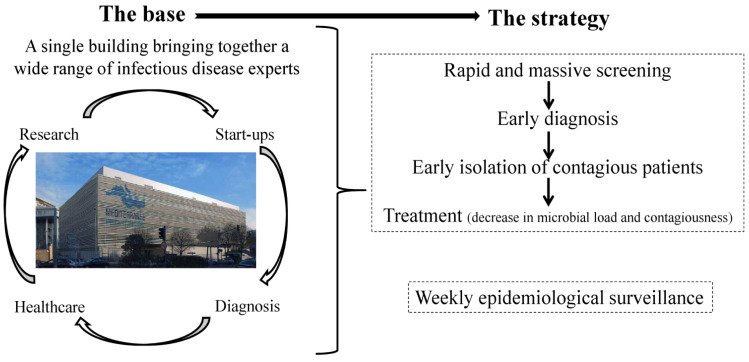
The base and the strategy of contagion management at the IHU.

The Méditerranée Infection University Hospital Institute (IHU) is located in a single building on the Marseille Medical Timone Campus in France and is entirely dedicated to infectious diseases (Figure 1) [1]. This modern building opened four years ago with the aim of treating contagious patients and dealing with health crises [1]. The building combines treatment, diagnosis, research, and start-ups dedicated to infectious diseases. The building as a whole is subject to strict card access control.

One key in improving the management of infectious diseases in the IHU has been to develop innovative tools such as epidemiological monitoring and the ability to mass screen the population. Weekly epidemiological monitoring, including surveillance of the microorganisms, detected in patients’ samples analysed by the IHU diagnostic laboratory, the numbers and types of samples received and of a panel of microorganisms identified in other public or private laboratories in the Provence Alpes Cote d’Azur area (South-East of France) is carried out [2]. This surveillance makes it possible to identify the occurrence of abnormal events and to detect potential health crises.

**Figure 2:**
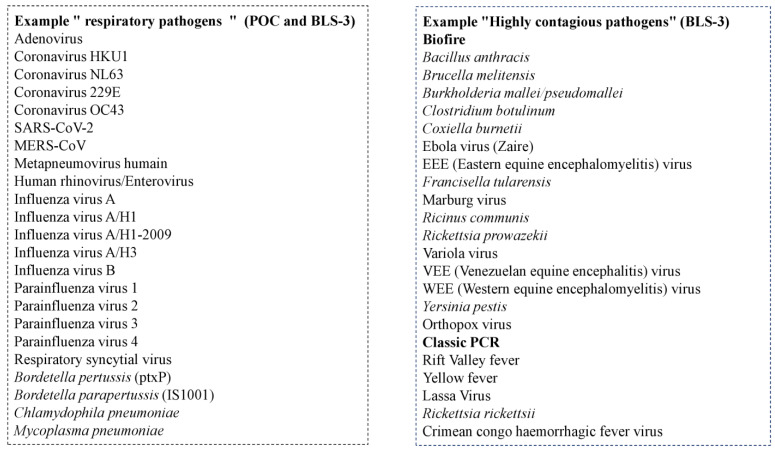
Examples of the large panel of microorganisms tested at the IHU.

The IHU strategy is also based on the rapid ability to carry out massive screening of people. Early diagnosis makes it possible to isolate contagious patients at an early stage and to initiate treatment to reduce the microbial load and contagiousness [3,4]. The key to rapid microbiological diagnosis is our Point-Of-Care laboratory (POC) [5,6]. The rapid tests are mainly based on real-time qPCR or immunochromatographic assays. All the equipment necessary for carrying out the analyses is contained in a small operational room situated in a strategic location in each of the IHU and emergency departments. Sampling and testing are performed using a syndromic approach based primarily on clinical manifestations. The POC laboratory influences patient treatment by answering three questions: (1) Is it necessary to isolate the patient? (2) Is it necessary to hospitalise the patient? (3) Is a specific treatment required? A large panel of microorganisms can be tested using a syndrome-based approach (Example “respiratory pathogens”) (Figure 2).

The rapid diagnosis of highly pathogenic infectious diseases is also performed in the biosafety level 3 laboratory (BSL-3) and in the POC laboratory of the BSL-3 hospital ward (Figure 2). Both laboratories are equipped with negative pressure in order to avoid the transmission of pathogens to the outside, and personal protective equipment (PPE) is mandatory and adapted to the assessed risk. Samples of infected or suspected patients are transferred to a level 3 biosafety cabinet, which contains the technology required for microbiological diagnosis. First, a molecular diagnostic automate (Biofire Filmarray, bioMérieux) can detect a large panel of agents (BIOFIRE^®^ RP2.1 plus panel and BioThreat Panel) in about 45 min. It is also possible to perform basic biology parameters, such as blood count, biochemistry, coagulation, blood groupings in collaboration with the French national blood agency, malaria rapid diagnostic tests, *Legionella* antigen urinary test, as well as basic microbiological diagnosis (urine analysis, blood cultures, antibiograms, etc.).

**Figure 3:**
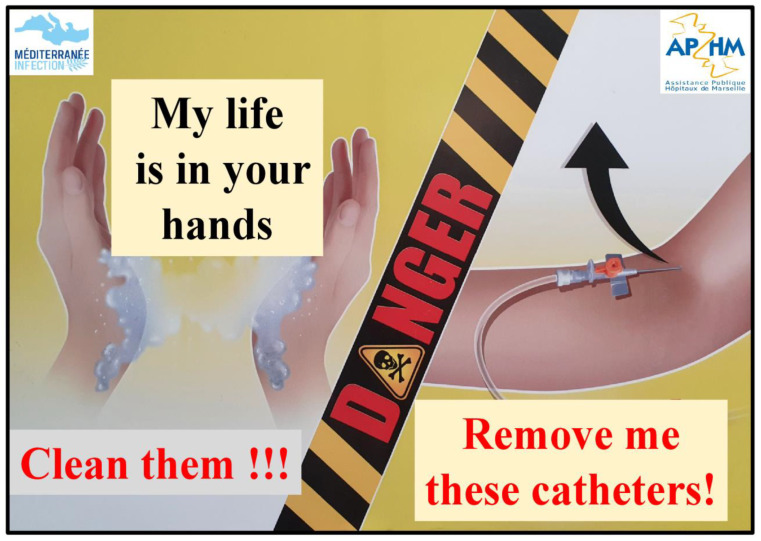
Display on bedroom doors, on the “healthcare” side.

For the management of infectious patients, the IHU has three hospitalisation units, each with 25 beds, one of which is divided into three modules in which negative pressure can be independently implemented [1]. All patients are accommodated in single rooms. There are two entrance doors for each bedroom, one for healthcare workers opening onto the internal corridor and one for family members opening onto the external corridor when the patient’s condition permits visits. There is a device in front of each room on the healthcare side that provides PPE and an alcohol-based solution dispenser at the entrance to each room. In the corridor for patients’ families, alcohol-based solution and protective mask dispensers are also available. Hand hygiene is another basis of contagion management at the IHU. There is wide access to alcohol based solutions throughout the building with nearly 600 dispensers available. IHU teams have long conducted hand hygiene monitoring and compliance studies, as well as awareness-raising campaigns and have adopted an anthropological approach to understanding healthcare provider behaviour towards hand hygiene protocols [7,8,9,10]. There is a sign on the doors of the rooms on the “healthcare” side that displays awareness-raising messages. “My life is in your hands... Clean them!!!” and “Remove those catheters!” to remind healthcare workers of the risks posed by medical devices and to reassess their need on a daily basis (Figure 3).

The IHU was, therefore, in theory, a modern institute to deal with contagious diseases and epidemics. The COVID-19 pandemic has confronted all healthcare facilities around the world with many challenges. We will see here point by point how, in practice, the IHU and its teams faced this epidemic.

## 2. Material and Methods

### 2.1. The Global Shortage of the First Wave of the COVID-19 Pandemic

During the first wave of the pandemic, which took place between 27 January and 14 June 2020, we had to organise the management of patients despite a major lack of PPE worldwide and lots of other types of equipment (masks, gloves, coveralls, gowns, aprons, glasses, visors, etc.) [11,12,13,14,15,16,17,18,19]. As early as January 2020, we ordered extra masks, but stocks were already running out. Stocks of alcohol-based solutions were also running out. It was difficult to stock up on reagents and equipment to perform molecular biology analyses to detect SARS-CoV-2, but also there was a shortage of swabs for conducting nasopharyngeal sampling. In accordance with our usual strategy and given the extent of the epidemic, we were also facing a massive influx of patients. We will describe the means implemented to overcome this global shortage.

### 2.2. Setting up Circuits

The various “highly contagious” circuits (patients, linen, waste, etc.) had been designed when the building was created and was thus applied to the COVID-19 epidemic. However, the patient circuits had to be adapted, and we will see how.

### 2.3. Massive Diagnosis Screening

The massive diagnosis screening required not only human but also technical reinforcements, which are described below.

### 2.4. Staff Screening for SARS-CoV-2

Workers of the Institute were screened for SARS-CoV-2 by RT-PCR using nasopharyngeal samples. In parallel, a SARS-CoV-2 serological assessment was performed. Staff members were also interviewed about the fear of being infected with SARS-CoV-2. All the results have been analysed by occupation.

### 2.5. Challenges between the Two Waves of the COVID-19 Pandemic

Careful measures were taken to avoid transmission of the virus in clinical wards receiving both COVID-19 and non- COVID-19 patients. We also had to face another massive influx of people coming to be tested at the IHU and had to regulate the flow of people to avoid a high concentration of patients queuing. There was also a need for reliable rapid tests.

## 3. Results

### 3.1. The Global Shortage of the First Wave of the COVID-19 Pandemic

Overall, during the first wave, 141,240 samples were tested, 3538 patients were followed up in the day hospital, and 702 patients were hospitalised according to the guidelines established for the management of COVID-19 in the IHU. We received donations from other research and diagnostic laboratories within the Aix-Marseille University as well as from Marseille public hospitals and French companies (PPE, materials, reagents, alcohol-based solutions, etc.). We disinfected and recycled coveralls. To do so, we adopted three strategies depending on various locations. For re-use in the BSL-3 laboratory, we decontaminated the coveralls using the airborne disinfection method with Bioquell Z2 and hydrogen peroxide (Bioquell HPV-AQ) or by autoclaving at 121 °C for 20 min. For other re-uses, a steam decontamination system was installed in a tent in the outdoor car park of the IHU. We also rationalised the use of PPE. Thanks to the private Méditerranée Infection foundation, which controls the IHU, we were able to be highly reactive in purchasing supplies, which was essential in a time when suppliers were running out of equipment. With regard to human resources, we received reinforcements from healthcare workers from other departments of the Hospitals of Marseille as well as from voluntary healthcare workers from the private sector. We also received logistical assistance from the Marseille firefighter service to help with triaging people for testing.

We also faced a global shortage of laboratory reagents and small equipment as well as uncertainty about their availability [20]. Other diagnostic and research laboratories in the city were initially able to help us by providing reagents and/or consumables from their own stock. As a national reference centre for rickettsiae, rickettsioses, and zoonotic diseases, we contacted veterinary laboratories that held reagent stocks but which had not been authorised for use in the diagnosis of human infections. Given the lack of specific swabs to obtain nasopharyngeal samples, we evaluated the situation and used faecal swabs until specific nasopharyngeal swabs were replenished.

### 3.2. Setting Up Circuits

To cope with the massive influx of patients, it was necessary to create areas to carry out massive patient screening, as well as consultation areas enabling the reception of a large number of patients. Thus, the reception hall was converted into a testing area. Low-dose thoracic CT scans were performed in the radiology department located 100 m from the IHU. Besides, there was no intensive care unit at the IHU, but an intensive care unit was located within 100 metres of the IHU. However, since September 2020, there has been an intermediate care unit where high-flow oxygen therapy can be performed on patients infected with SARS-CoV-2 and who were not eligible for transfer to an intensive care unit (elderly patients and/or those with severe comorbidities) [21]. Finally, there was no specific area for patients who had been admitted with suspected COVID-19 but had received a negative PCR result.

### 3.3. Massive Diagnosis Screening

The largest number of tests performed in one day was 3809, with a maximal capacity of 5000 tests per day. Overall, 20 automated nucleic acid extractors with a capacity of 14 to 96 samples each and 16 thermal cyclers with a capacity of 96 samples each were available in the Institute at the onset of the epidemic. To increase the diagnostic yield as well as to cope with reagent shortage, together with maintaining the other diagnostic activities, four KingFisher extractors (96 samples in 40 min), one PerkinElmer extractor (96 samples in 90 min), one QIAcube extractor (96 samples in two hours), one MGI extractor (96 samples in 90 min), two Light Cycler 480 thermal cyclers (96 samples in two hours) and three NeuMoDx molecular (extraction and PCR) thermal cycler systems (96 samples in two hours) were acquired between March and June 2020. In addition, 16 VitaPCR thermal cyclers (Credo Diagnostics Biomedical) were also purchased for rapid molecular screening (each performing one test every 20 min). This multiplication of the PCR systems was imperative, not only to be able to analyse an increasing number of samples, but also to cope with delayed deliveries and stockouts of reagents and in order to have devices that enabled the fastest testing for emergencies while maintaining high throughput analysis capability. Daily briefings were completed with the management team regarding the molecular diagnosis of COVID-19. These briefings included an update on the analyses (number of tests carried out the day before, problem of interpretation and reporting of results to patients or their physicians, deadline for reporting results, etc.), stocks of reagents and small equipment (capacity for analyses to be carried out with the available stocks, orders in progress and delivery times, suppliers to follow-up, orders to be placed), human resources (number of technicians present and trained to ensure diagnostic continuity 24 h a day, seven days a week) as well as on the various adjustments made with the new equipment and reagents.

### 3.4. Staff Screening for SARS-CoV-2

During the first wave, we screened healthcare workers of the Institute who were in direct contact with patients every two days by RT-PCR [22] (Table 1). During the second wave, this was performed once or twice a week. At the end of the first wave (end of April-Beginning of May 2020), a SARS-CoV-2 serological assessment of 488 IHU staff members was performed (Table 1) [23,24]. A total of 22 were positive (4.5%), including 6 nurses, 3 housekeepers, 3 physicians, 2 nursing assistants, 2 medical fellows, 2 health executives, 2 administrative staff, and 1 logistician. In mid-December 2020, another SARS-CoV-2 serological assessment of 286 IHU staff members was performed (Table 1). A total of 46 were positive (16%), including 15 nurses, 8 administrative staff, 5 physicians, 4 laboratory technicians, 4 housekeepers, 3 health executives, 3 nursing assistants, 2 engineers, 1 pharmacist, and 1 researcher/PhD student. If we compile the data from the first and second waves, 61 staff members out of 656 (9.3%) were infected by SARS-CoV-2.

For administrative staff, contamination occurred outside the IHU, except for four who were contaminated by other staff with whom they shared an office, and they had been contaminated in the community. For the engineers, pharmacist, researcher/PhD student, and laboratory technicians, contamination occurred outside the IHU. Concerning medical fellows and physicians, the contamination occurred a priori at the IHU. For nurses, nursing assistants, housekeepers, and health executives, although during the first wave most contaminations occurred a priori at work, this was not the case during the second wave.

The 286 IHU staff members were also interviewed about the fear of being infected with SARS-CoV-2, and 282 answers were obtained (Table 2). Most of them (178; 63%) declared “no fear at all” of being infected with SARS-CoV-2, 25 (9%) “a little”, 44 (16%) “moderate”, and 35 (12%) “great fear”. With the exception of one person who self-medicated with high doses of corticosteroids, no serious form requiring hospitalisation was observed among the staff. In addition, in the BSL-3 laboratory, 7112 samples were inoculated for SARS-CoV-2 cultures. Of them, 3070 were positive. No contamination was observed among the BSL-3 staff.

### 3.5. Challenges between the Two Waves of the COVID-19 Pandemic

**Figure 4:**
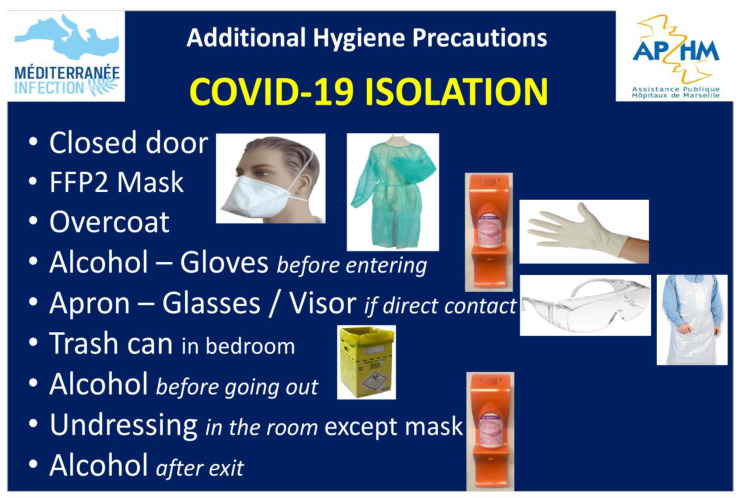
Specific sign placed on the doors of COVID-19 patient rooms.

A specific sign was placed on the doors of COVID-19 patient rooms listing the main protection measures in clinical wards receiving both COVID-19 and non- COVID-19 patients (Figure 4). To cope with the new massive influx of people coming to be tested at the IHU, in addition to the possibility of turning up without an appointment, we organised a queue for patients who had made an appointment via the internet, with a capacity of approximately 700 appointments per day (excluding Saturdays and Sundays), and reaching a capacity of 1000 at the peak of the outbreak. We also deployed a rapid registration system (SI-DEP) that took three minutes per patient. For this, 14 administrative staff members were recruited to speed up patient registration and to communicate with them.

We evaluated antigenic tests as well as a rapid molecular test, the VitaPCR SARS-CoV-2 [25,26]. We first demonstrated the lack of sensitivity of antigen tests and also the reliability of the VitaPCR assays. By collaborating with one of the start-ups hosted at the IHU, we installed these PCR machines in two tents in the reception hall of the IHU, right next to the entrance to the COVID-19 consultation facility, in order to be able to safely sample patients and obtain results in just over 20 min. As a single device can test only one sample at a time, we deployed 16 devices, including 6 in the tents, 6 in the 2 POC laboratories (those located in the IHU and those in North Hospital) and 4 in a newly created laboratory in the Timone hospital emergency ward in order to be able to diagnose people presenting to the emergency room as quickly as possible.

## 4. Discussion

The COVID-19 outbreak prompted us to move from theory to practice. The biggest lesson from the first wave was the need to have stocks of PPE, materials, and reagents sufficient to cope with a shortage due to a global health crisis. It is in this context that at the end of the first wave, we continued to equip ourselves and build up reagents and PPE stocks and to transform meeting rooms and common areas into storage areas as reserves were already filled and into laboratories.

The lack of infection among the BSL-3 staff confirmed that most risks from biological hazards can be reduced through the use of appropriate procedures and techniques, adequate equipment and infrastructure and staff training and that human error is significantly involved in staff contamination [27].

Between 27 January 2020 and 5 January 2021, 434,925 samples were tested for SARS-CoV-2, 12,055 patients with COVID-19 were followed up in the day clinic, and 1888 patients were hospitalised at the IHU according to our guidelines. Regarding nosocomial infections, no catheter-related bloodstream infections and no outbreaks of multidrug-resistant bacteria were observed. After more than three days of hospitalisation, a respiratory superinfection was identified in five patients, including two with *Streptococcus pneumoniae*, two with *Candida albicans*, and one with both *Candida tropicalis* and *Staphylococcus aureus*.

Finally, by constantly adapting in order to be able to comply with our strategy, the IHU managed to cope with the various stockouts and the massive influx of patients. The COVID-19 epidemic made it possible to expand and upgrade its stock of equipment and to improve patient circuits and flows to better manage infected patients.

## Figures and Tables

**Table 1:** Staff screening of the Méditerranée Infection University Hospital Institute (IHU) for COVID-19 in the first and second waves.

	First Wave			Second Wave		
IHU Staff		23 People with a Previous Positive SARS-CoV-2 PCR			48 People with a Previous Positive SARS-CoV-2 PCR	
	488 People Tested by Serology	Positive Serology (%)	Negative Serology	286 People Tested by Serology	Positive Serology (%)	Negative Serology or Not Performed
Administrative staff members	51	2 (4%)	0	25	8 (32%)	0
Engineers	25	0	0	28	2 (7.1%)	0
Health executives	13	2 (15.4%)	0	8	3	0
Housekeepers	25	3 (12%)	0	14	4 (28.5%)	1
Laboratory technicians	102	1 (1%)	0	47	4 (8.5%)	0
Logisticians	21	1 (4.8%)	0	2	0	0
Medical fellows	32	2 (6.2%)	0	20	0	0
Nurses	93	6 (6.5%)	1	36	15 (41.6%)	0
Nursing assistants	33	2 (6%)	0	15	3 (20%)	1
Pharmacists	2	0	0	7	1	0
Physicians	48	3 (6.2%)	0	23	5 (21.7%)	0
Researchers/PhD students	35	0	0	60	1 (1.7%)	0
Porters	8	0	0	1	0	0
Total	488	22 (4.5%)	1	286	46 (16%)	2

**Table 2:** Screening of 286 IHU staff members on their fear of being infected with SARS-CoV-2.

Fear of Being Infected with SARS-CoV-2	Number of Staff Members	Results by Occupation	Results for Infections with SARS-CoV-2
Great	35	5 administrative staff members	0 administrative staff members
		5 engineers	
		1 health executive	
		3 housekeepers	1 housekeeper
		3 medical fellows	
		4 nurses	1 nurse
		2 nursing assistants	
		1 pharmacist	
		2 physicians	
		9 researchers/PhD students	1 researcher/PhD student
Moderate	44	4 administrative staff members	2 administrative staff members
		8 engineers	
		1 health executive	
		1 housekeeper	
		11 laboratory technicians	
		3 nurses	1 nurse
		1 pharmacist	
		15 researchers/PhD students	
ittle	25	1 administrative staff member	1 administrative staff member
		3 engineers	
		1 housekeeper	
		1 laboratory technician	
		3 medical fellows	
		3 nurses	2 nurses
		1 nursing assistant	
		4 physicians	1 physician
		7 researchers/PhD students	
		1 porter	
No fear at all	178	15 administrative staff members	4 administrative staff members
		12 engineers	2 engineers
		4 health executives	2 health executives
		7 housekeepers	3 housekeepers
		36 laboratory technicians	4 laboratory technicians
		2 logisticians	
		14 medical fellows	
		26 nurses	12 nurses
		12 nursing assistants	3 nursing assistants
		5 pharmacists	1 pharmacist
		17 physicians	4 physicians
		28 researchers/PhD students	
No answer	4	2 housekeepers	2 housekeepers
		1 health executive	1 health executive
		1 researcher/PhD student	

## Data Availability

All data are available upon request to the corresponding author.
